# Electronic properties of several two dimensional halides from ab initio calculations

**DOI:** 10.3762/bjnano.10.82

**Published:** 2019-04-03

**Authors:** Mohamed Barhoumi, Ali Abboud, Lamjed Debbichi, Moncef Said, Torbjörn Björkman, Dario Rocca, Sébastien Lebègue

**Affiliations:** 1 Laboratoire de la Matière Condensée et des Nanosciences (LMCN), Université de Monastir, Département de Physique, Faculté des Sciences de Monastir, Avenue de l’Environnement, 5019 Monastir, Tunisia; 2 Laboratoire de Physique et Chimie Théoriques (LPCT, UMR CNRS 7019) Institut Jean Barriol, Université de Lorraine, BP 239, Boulevard des Aiguillettes 54506 Vandoeuvre-lès-Nancy, France; 3 Graduate School of Energy, Environment, Water, and Sustainability (EEWS), Korea Advanced Institute of Science and Technology (KAIST), Yuseong-gu, Daejeon 305-701, Korea; 4 Physics/Department of Natural Sciences, Åbo Akademi University, Porthansgatan 3, 20500 Turku, Finland

**Keywords:** density functional theory, electronic properties, halide monolayers

## Abstract

Using density functional theory, we study the electronic properties of several halide monolayers. We show that their electronic bandgaps, as obtained with the HSE hybrid functional, range between 3.0 and 7.5 eV and that their phonon spectra are dynamically stable. Additionally, we show that under an external electric field some of these systems exhibit a semiconductor-to-metal transition.

## Introduction

The discovery of graphene [1] by exfoliation [2] opened a new era in several domains of science. Graphene has attracted great attention due to its unique properties [3] and because it offers many advantages in comparison with more common materials [4–7]. Although graphene is the most extensively studied 2D crystal [8], graphene is gapless, and this lack of a bandgap hampers its application in electronic and optoelectronic devices. This has motivated the research on other two-dimensional (2D) materials with a finite bandgap, such as transition-metal dichalcogenides (TMDs) [9], phosphorene [10–11], and hexagonal boron nitride (h-BN) [8], among others [12–13], which are suitable for applications in electronic and photonic devices. However, in order to improve performance and possibly access new properties, the quest for new 2D compounds is an active research field [14–16]. A family of layered materials that attracted interest over the past few years are halides. For instance, BiOX (with X = Cl, Br and I) compounds are known to be promising photocatalysts [17–19]. In this context, Sharma et al. [20] have studied the synthesis and the photocatalytic properties of BiOX compounds under three different exposure conditions. Also, transition-metal oxychlorides MOCl (M = Sc, Ti, V, Cr, Fe) systems possess interesting electronic and magnetic properties [21–24]. Bismuth oxyhalides have been investigated as catalysts, ferroelectric materials, storage materials, and pigments [25]. Siidra et al. [26] have investigated the synthesis and modular structural architectures of mineralogically inspired novel Pb oxyhalides. In parallel, theoretical works about these compounds were realized. For example, first-principles calculations with density functional theory (DFT) [27] were conducted to study the physical and chemical properties of bulk BiOX compounds as a complement to experiments [28]. In 2006, Zhang et al. [17] calculated the electronic structure of bulk BiOCl with the tight-binding linear muffin-tin orbital (TB-LMTO) code and the local density approximation. Bell and Dines [29] reported the results of DFT studies on the geometries and vibrational spectra of several chromium oxo-anions and bulk oxyhalides. Ruckamp et al. [21] reported on the magnetic, thermodynamic and optical properties of the quasi one-dimensional quantum antiferromagnets TiOCl and TiOBr. Durig et al. [30–31] have investigated the vibrational properties and Raman intensities (RI) of several bulk oxyhalides CrOX (where X = F, Cl) using ab initio calculations. Additionally, Zhang et al. [32] have studied the stability of bulk BiOX compounds by performing phonon calculations. These studies were conducted on bulk materials but little is known on the structural and electronic properties of the corresponding isolated layers. By using a datamining procedure some halides were identified as possible 2D materials [14] but their dynamical stability and detailed electronic properties were not studied. In the present work, using density functional theory, we investigate the structural, vibrational and electronic properties of several 2D halide compounds such as the bromides (XOBr and X′FBr with X = Ac, Bi; X′ = Ba, Ca), the fluorides (XOF with X = Cr, Ga, In, La), the chlorides (XOCl and X′FCl with X = Ac, Al; X′ = Ba, Bi), and the iodides (XOI with X = Bi, La, Sc, Y).

## Computational Details

Our present investigation of the electronic properties of the 2D halides employs density functional theory (DFT) as implemented in the Vienna ab initio simulation package (VASP) [33–34]. We used the generalized gradient approximation (GGA) [35] for the exchange–correlation functional, the projector augmented wave method [36], and a kinetic energy cutoff of 500 eV for the plane-wave basis set. For Brillouin zone (BZ) integrations, a mesh of 12 × 12 × 1 k-points [37] was used. Similar parameters were employed for hybrid Heyd–Scuseria–Ernzerhof (HSE) calculations [38], that were performed to obtain accurate values for the bandgap. Sufficient spacing (more than 17 Å) was put between the monolayers to avoid significant interactions between the periodically repeated images. Geometries (cell and atom positions) were relaxed with a threshold on forces of 10^−6^ eV/Å. The phonon dispersion curves of the single layers, as presented in Supporting Information File 1, were determined with the PHONOPY code [39] with a 5 × 5 × 1 supercell using density functional perturbation theory (DFPT) [40].

## Results and Discussion

In this section we discuss the structural and electronic properties of the compounds investigated in this paper. We will focus on XOBr and X′FBr (where X = Ac, Bi and X′ = Ba, Ca) for the bromides, on XOF (where X = Cr, Ga, In, La) for the fluorides, on XOCl and X′FCl (where X = Ac, Al and X′ = Ba, Bi) for the chlorides, and on XOI(X = Bi, La, Sc, and Y) for the iodides.

### Structural properties

**Figure 1:**
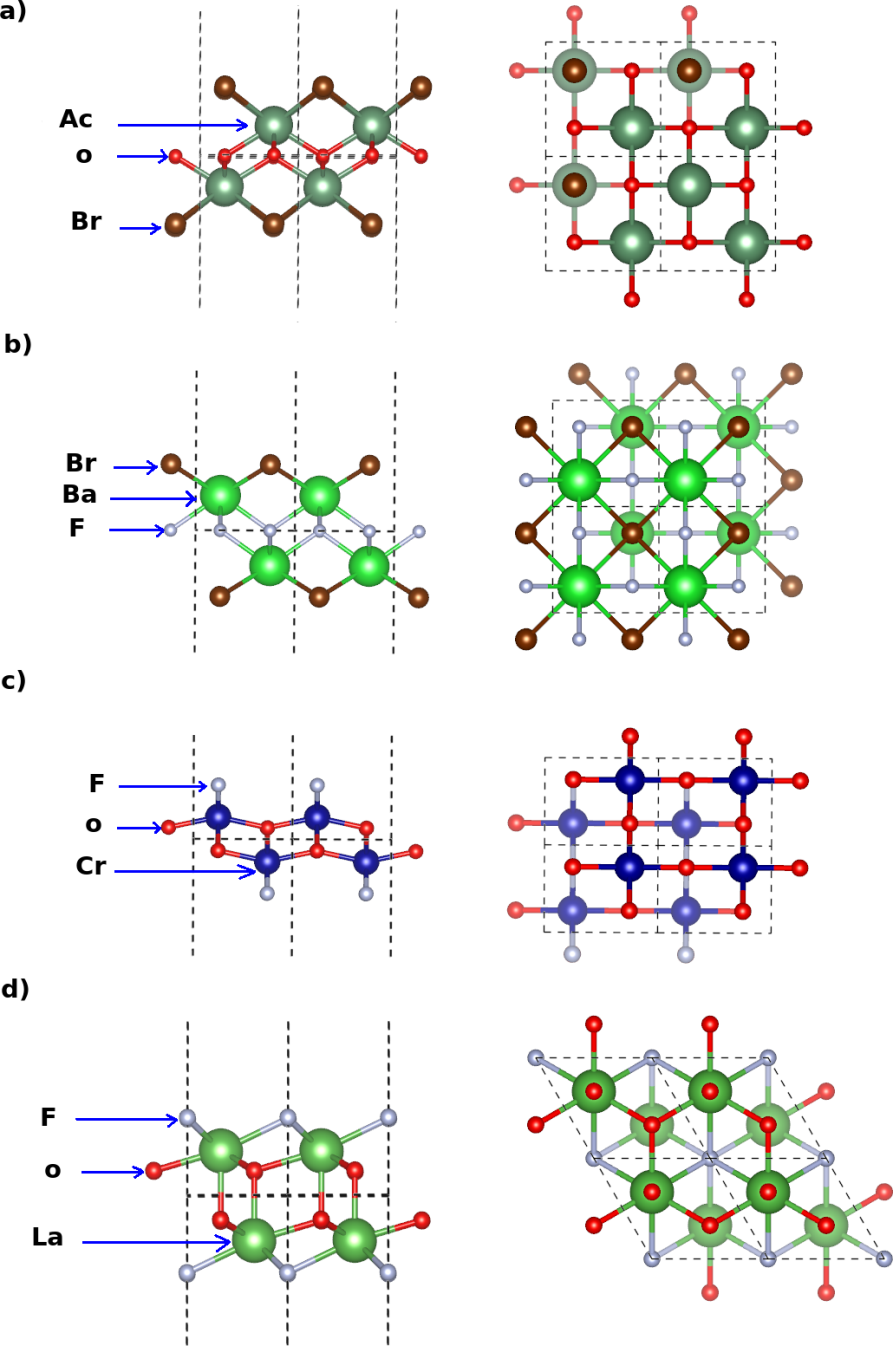
Side and top views of the crystal structures of a monolayer of: (a) AcOBr, (b) BaFBr, (c) CrOF and (d) LaOF.

The structure of the bromide (AcOBr and BaFBr) and of the fluoride monolayers (CrOF and LaOF) are shown in Figure 1. The structures of BiOBr and CaFBr are analogous to the one of AcOBr and the structures of GaOF and InOF are analogous to the one of CrOF. Accordingly, the corresponding geometries are not shown in Figure 1.

**Figure 2:**
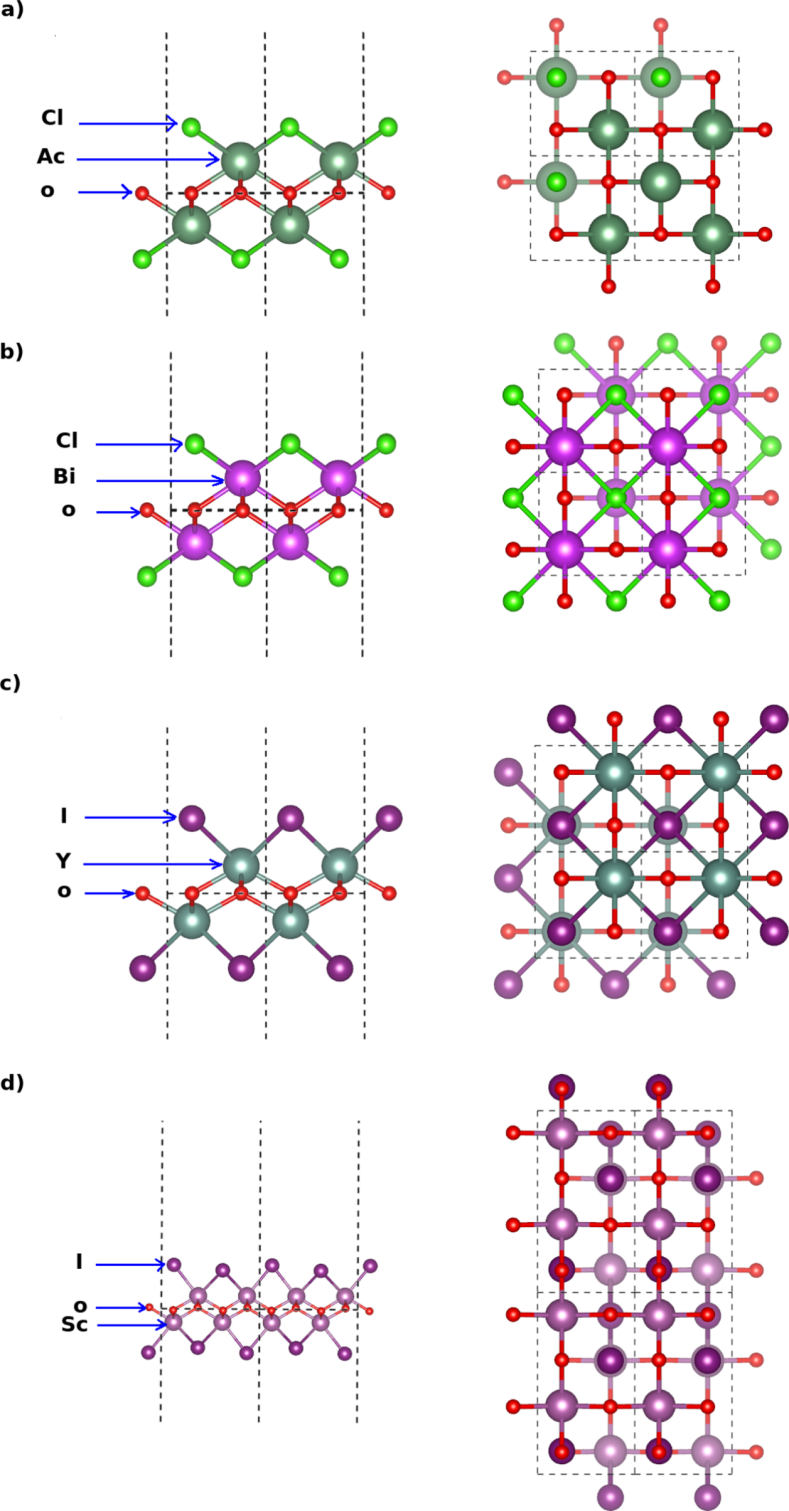
Side and top views of the crystal structure of a monolayer of: (a) AcOCl , (b) BiOCl, (c) YOI and (d) ScOI.

In the same way, in Figure 2, we show the crystal structures of the chloride and iodide monolayers (AcOCl, BiOCl, YOI, and ScOI). The geometries of AlOCl and BaFCl (not shown) are similar to the geometry of AcOCl. In Figure 2, we present also the structures of the iodide monolayers YOI and ScOI (the structures of BiOI and LaOI are similar to the one of YOI). It can be noticed that each monolayer has a thickness of five atoms with sublayers formed by each chemical element.

The optimized lattice constants of all the monolayers are presented in Table 1 and compared with the available experimental data [41] for the bulk crystals. Our calculated lattice parameters are in good agreement with experiments. The slight difference can be traced back to the fact that the PBE functional is usually overestimating equilibrium lattice parameters (although the opposite is seen for BiOCl), to the fact that experiments are not done at *T* = 0, or to the fact that we are comparing data of bulk structures while our calculation are performed on monolayers, which can induce a small change in the value of the in-plane lattice parameters. Overall, the structures of the isolated layers are expected to be close to the structures of the layers in the corresponding bulk material.

**Table 1:** Comparison of our calculated (PBE) lattices constants (Å) with the experimental (E) values of the bulk structures [41].

	bromide monolayers				fluoride monolayers			
	AcOBr	BaFBr	BiOBr	CaFBr	CrOF	GaOF	InOF	LaOF

*a*^PBE^	4.190	4.365	3.970	3.976	3.890	3.795	4.139	3.975
*b*^PBE^	4.190	4.365	3.970	3.976	3.010	2.980	3.334	3.975
*a*^E^ [41]	—	—	3.916	—	—	—	—	—
*b*^E^ [41]	—	—	3.916	—	—	—	—	—

	chloride monolayers				iodide monolayers			
	AcOCl	AlOCl	BaFCl	BiOCl	BiOI	LaOI	ScOI	YOI

*a^PBE^*	4.150	3.178	4.289	3.875	4.033	4.105	7.241	3.900
*b^PBE^*	4.150	3.675	4.289	3.875	4.033	4.105	3.870	3.900
*a^E^* [41]	—	—	—	3.891	3.985	—	—	—
*b^E^* [41]	—	—	—	3.891	3.985	—	—	—

Also, we have checked the dynamical stability of the 2D structures by calculating their phonon spectra (see Supporting Information File 1). There are no imaginary frequencies, and therefore the compounds are dynamically stable in the form of a 2D layer.

### Electronic properties

**Figure 3:**
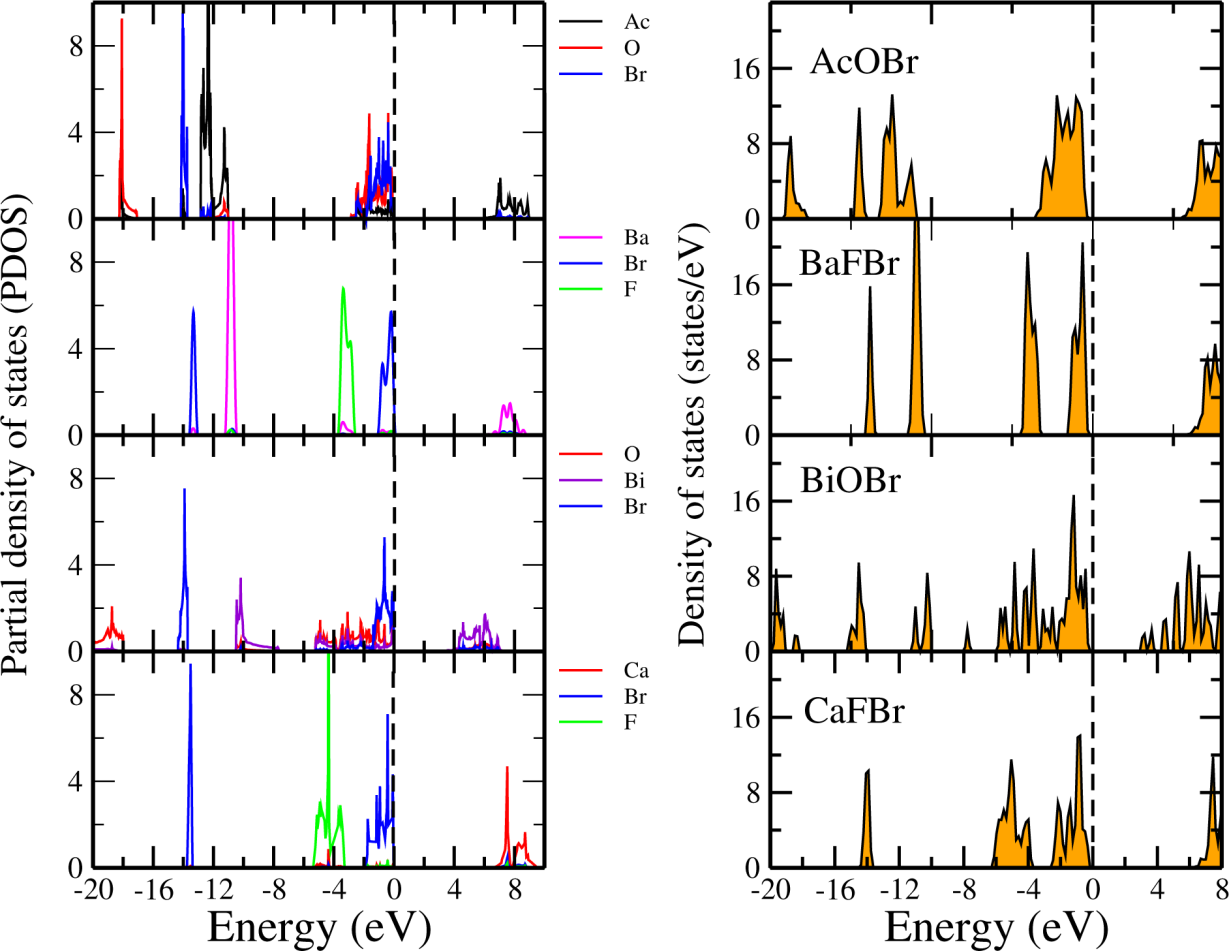
Partial and total density of states of monolayers of AcOBr, BaFBr, BiOBr, and CaFBr. The Fermi level is set to 0 eV.

In this section we discuss the electronic structure of the different monolayers. The total densities of states (TDOSs) and partial densities of states (PDOSs) obtained with the HSE functional for the different bromide monolayers are presented in Figure 3. Although the TDOSs are quite different from one compound to another (see the right part of Figure 3), for all of them the bromine atoms contribute significantly to the top of the valence bands. For instance, for AcOBr, the top of the valence bands is made of states coming from Ac, O, and Br, while Ac-derived states constitute most of the bottom of the conduction bands, as seen in the corresponding PDOS.

**Figure 4:**
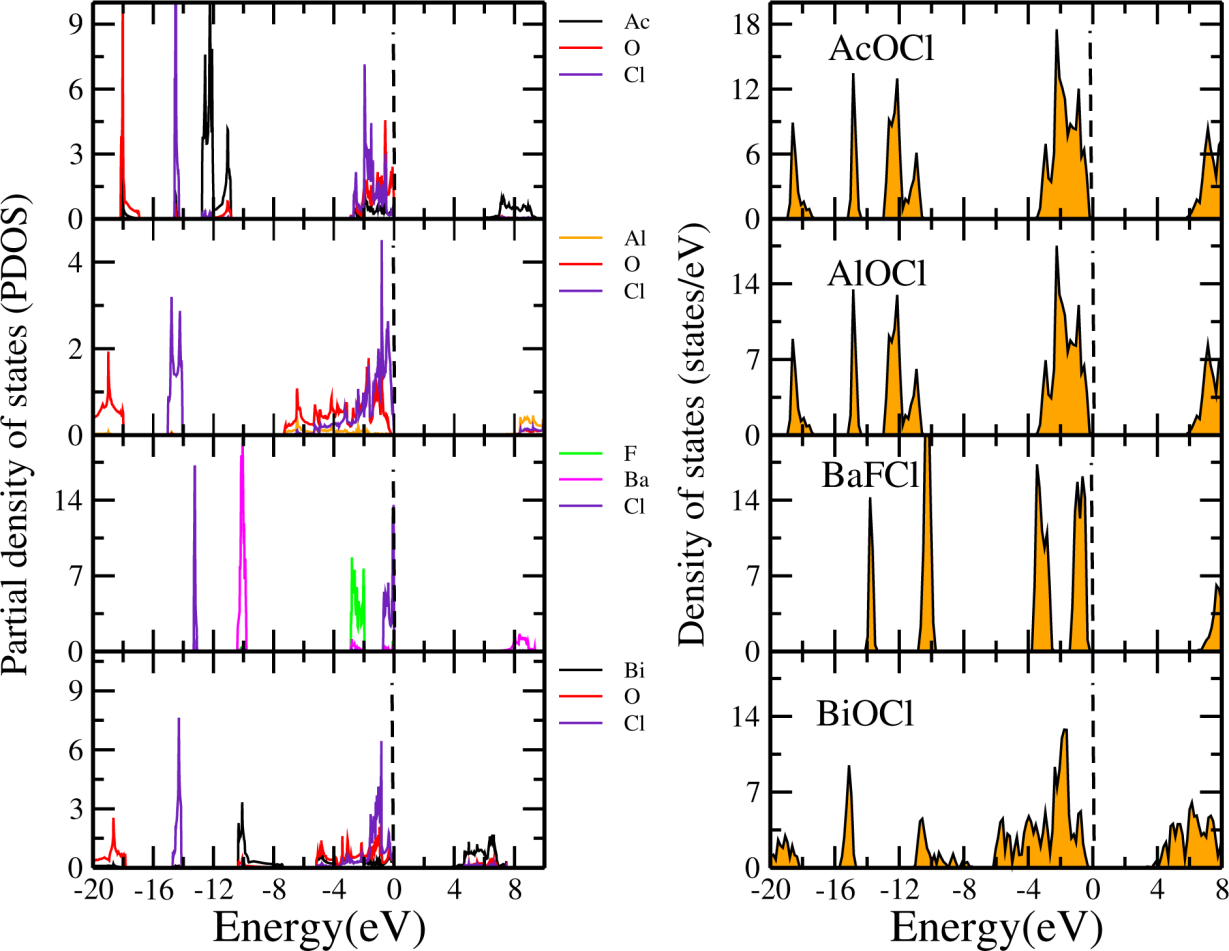
Partial and total density of states of monolayers of AcOCl, AlOCl, BaFCl, and BiOCl. The Fermi level is set to 0 eV.

**Figure 5:**
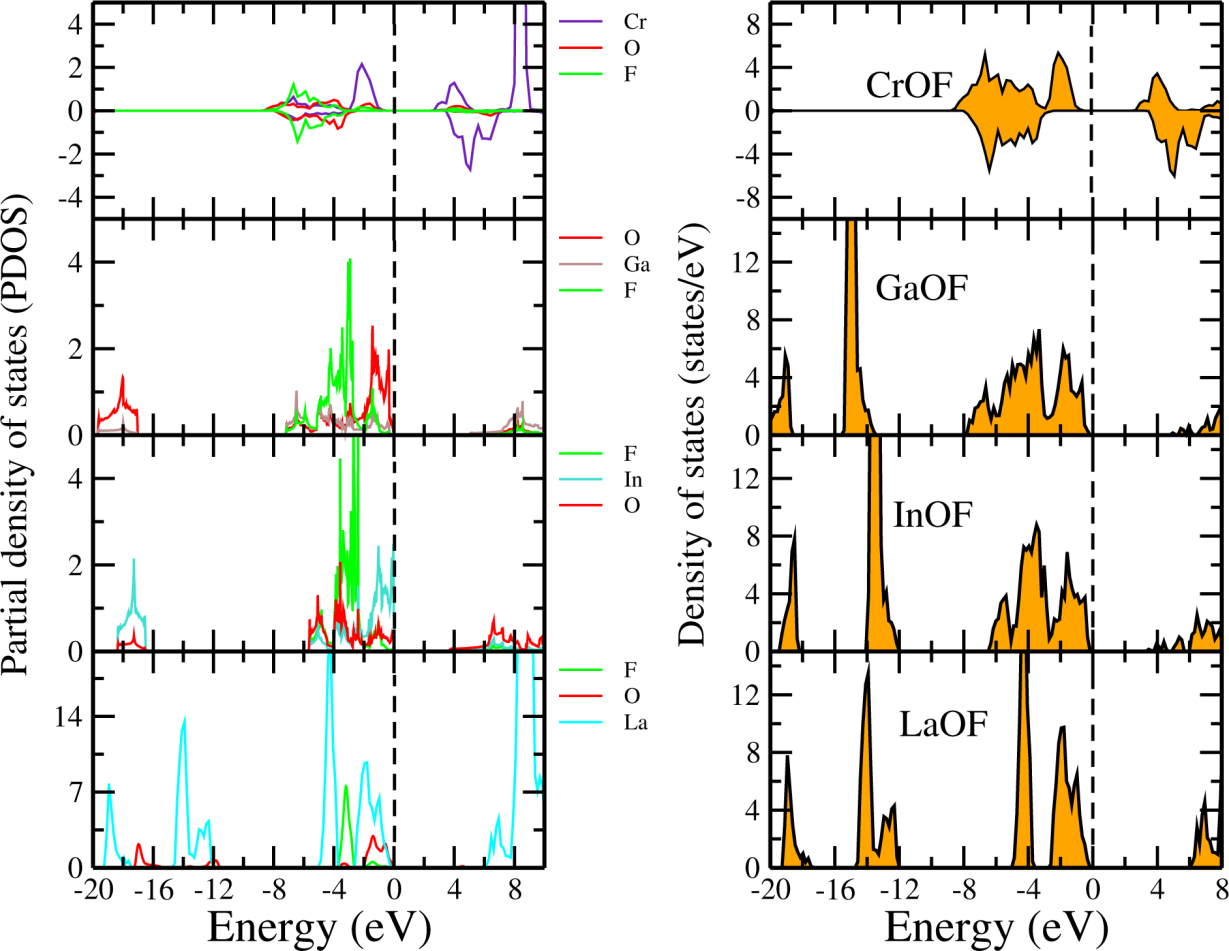
Partial and total density of states of monolayers of CrOF, GaOF, InOF, and LaOF. The Fermi level is set to 0 eV.

Similarly, we have computed the DOSs and PDOSs of XOCl (where X = Cr, Ga, In, La) monolayers, as presented in Figure 4. For instance, we can see from the PDOS of AcOCl that the valence bands come from the Ac, O, and Cl atoms while the bottom of the conduction bands correspond mainly to states derived from the Ac atoms. The DOSs and PDOSs of the fluoride monolayers CrOF, GaOF, InOF and LaOF are shown in Figure 5. From the DOS, it can be seen that the CrOF monolayer has a finite spin polarisation, and its magnetic moments are ordered ferromagnetically. Also, it is seen that the fluorine atoms contribute to the valence bands for all fluorides monolayers, while the bottom of the conduction bands is a hybridization between the Cr (La, Ga or In) and the O atoms.

**Figure 6:**
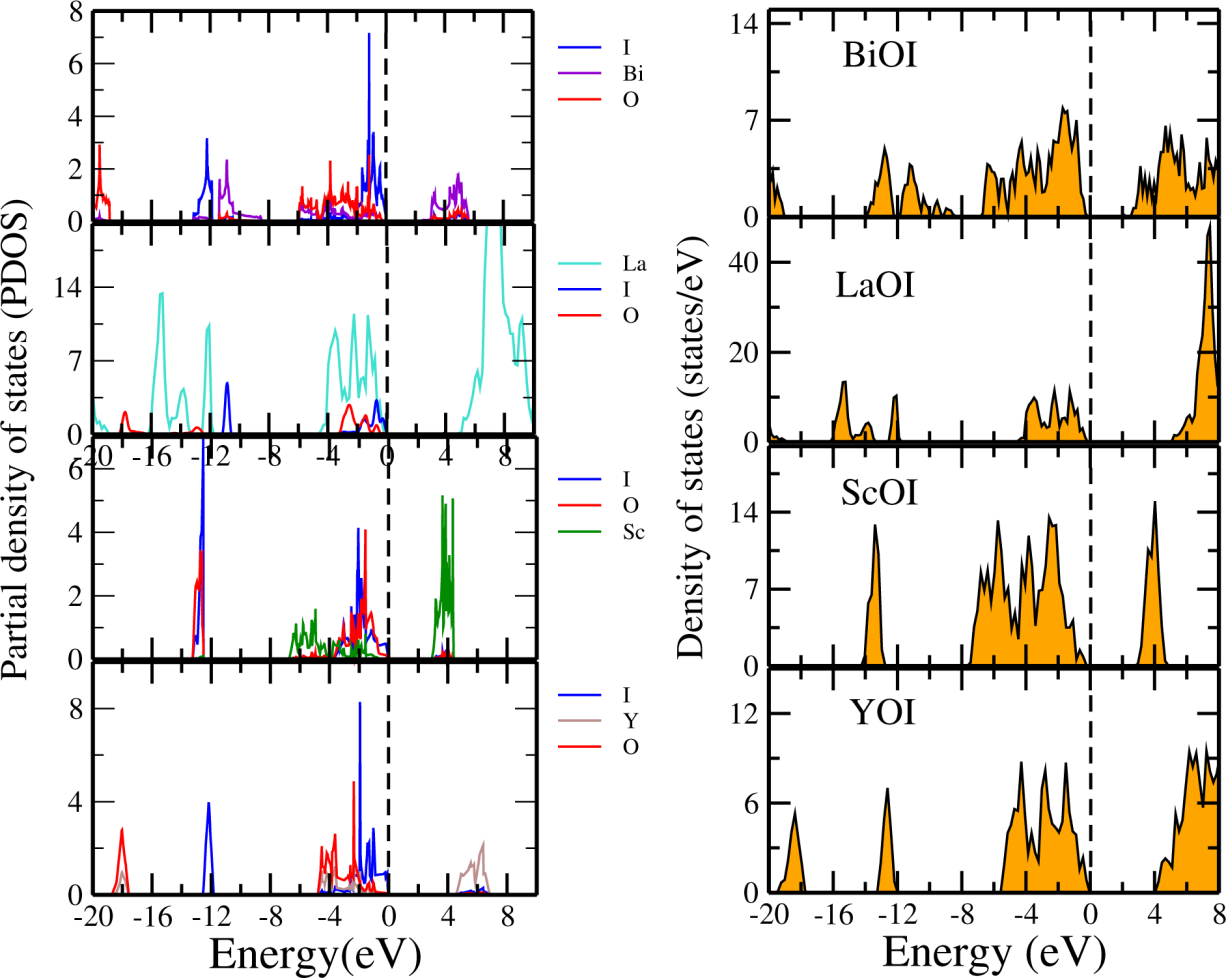
Partial and total density of states of monolayers of BiOI, LaOI, ScOI and YOI. The Fermi level is set to 0 eV.

We have performed a similar study in the case of the iodide monolayers. The corresponding DOSs and PDOSs of BiOI, LaOI, ScOI, and YOI are shown in Figure 6. For all the systems, we found that the valence band is mostly dominated by states of O and I. While the conduction band bottom mainly derives from the states of Bi for BiOI, La for LaOI, Sc for ScOI and Y for YOI.

**Figure 7:**
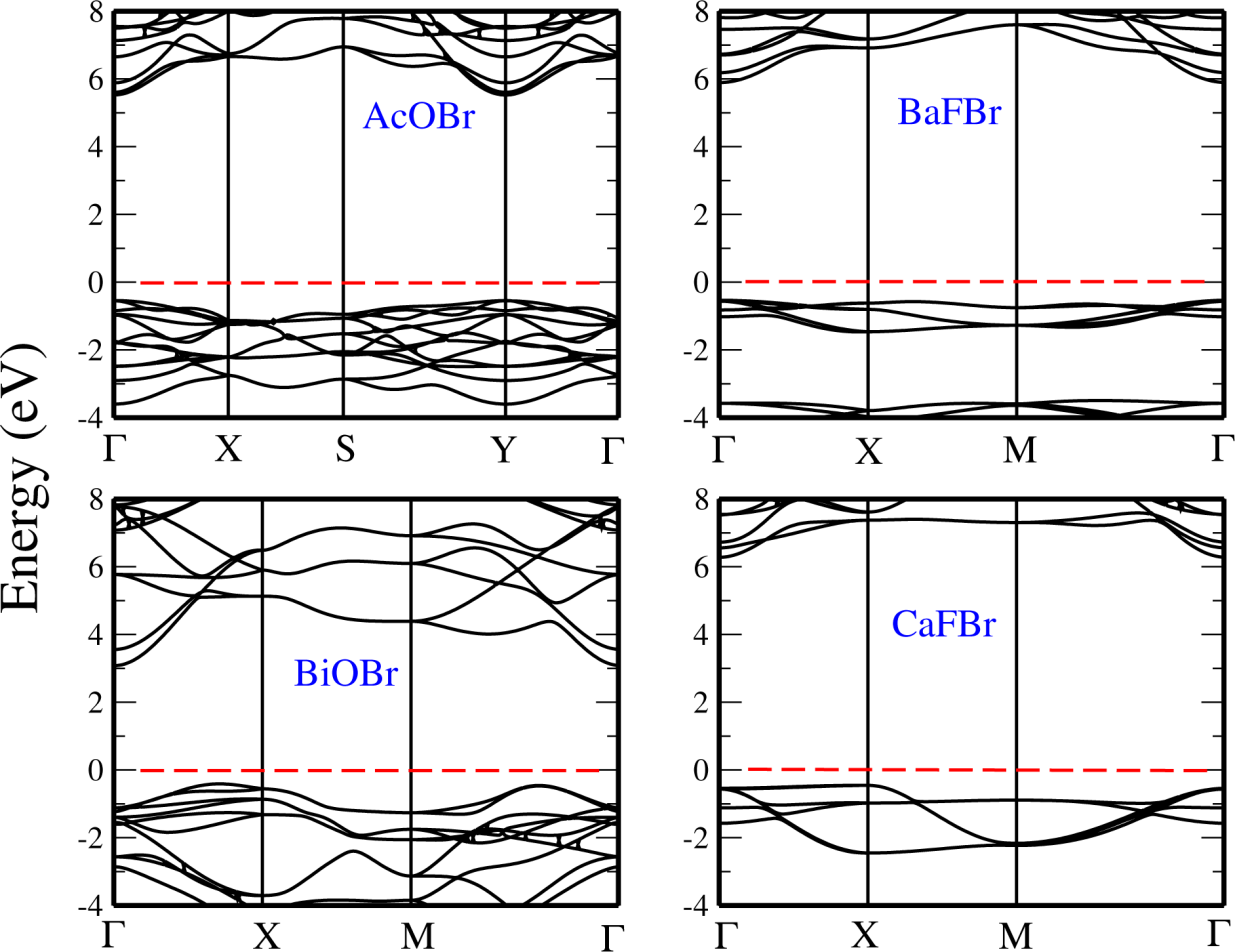
Band structures of monolayers AcOBr, BaFBr, BiOBr, and CaFBr, using the HSE functional. The Fermi level is set to 0 eV.

In addition, we have computed the electronic band structures of the various monolayers along high-symmetry directions in the Brillouin zone. The resulting band structures using HSE are presented in the following paragraphs. In Figure 7, we present the band structures of the AcOBr, BaFBr, BiOBr, and CaFBr monolayers. We found that the AcOBr, BaFBr, and CaFBr monolayers are direct-bandgap semiconductors with the valence-band maximum (VBM) and the conduction-band minimum (CBM) both located at the Γ point. The bandgap energies of these three compounds computed using the GGA functional are 4.57, 5.02 and 5.17 eV, respectively, while, when HSE is considered, the calculated bandgap energies are 6.1 eV for AcOBr, 6.6 eV for BaFBr, and 6.9 eV for CaFBr. In contrast to the other bromides, the BiOBr monolayer exhibits an indirect bandgap with the CBM located at the Γ point and the VBM located along the M–Γ line. Using the GGA, the bandgap of BiOBr is found to be 2.68 eV, which is increased to 4.50 eV when the HSE functional is used.

**Figure 8:**
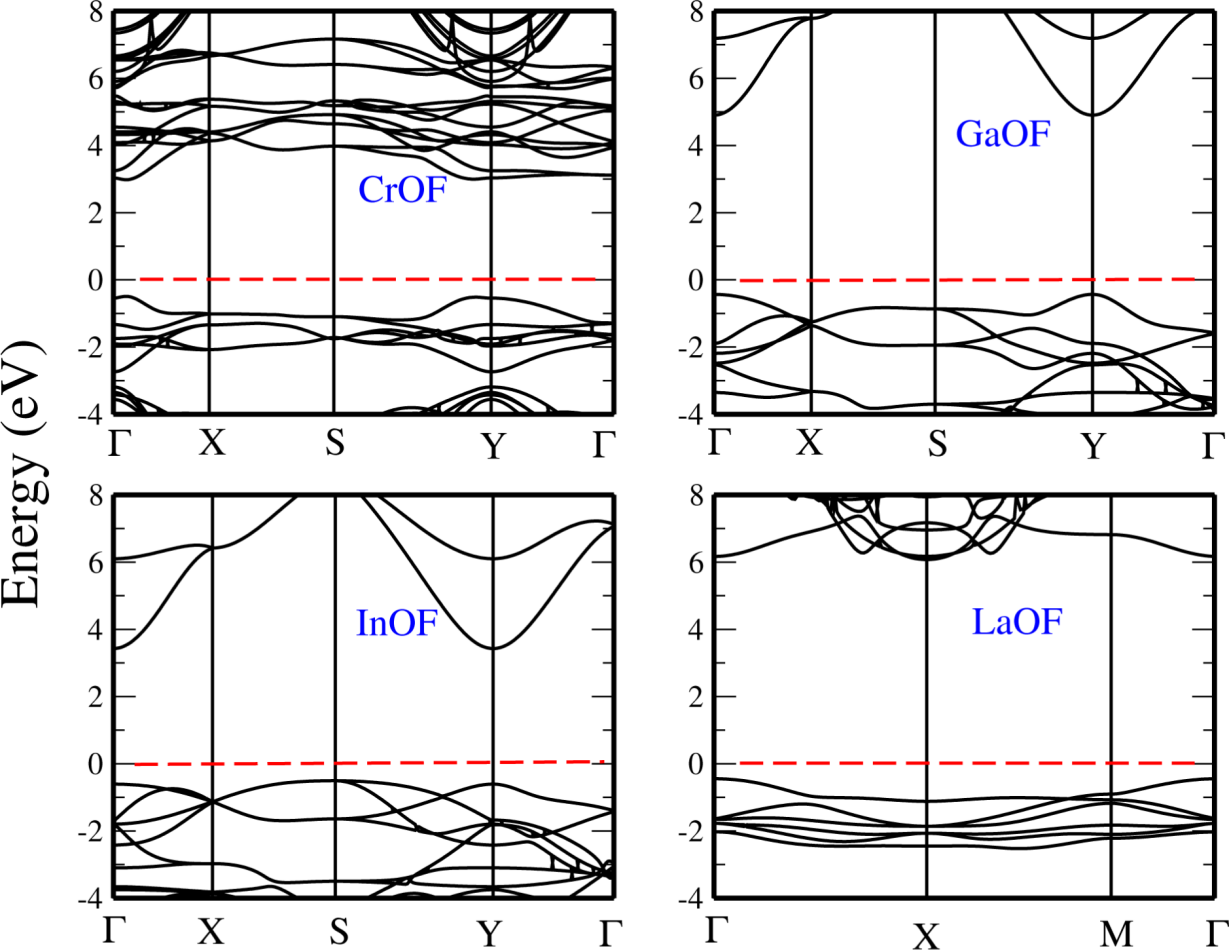
Band structures of the monolayers CrOF, GaOF, InOF, and LaOF, using the HSE functional. The Fermi level is set to 0 eV.

The HSE electronic band structures of the XOF monolayers (where X = Cr, Ga, In, La) are displayed in Figure 8. We observe that the bandgap of GaOF is direct, at the Γ point. Also in this case, the HSE approximation leads to significant changes in comparison with GGA. The bandgap increases from 3.07 eV (GGA) to 5.3 eV (HSE). The bandgap of InOF and LaOF are also found to be direct. With HSE InOF has a bandgap of 4.0 eV at the Y point, while LaOF has a bandgap of 6.0 eV at the Γ point.

**Figure 9:**
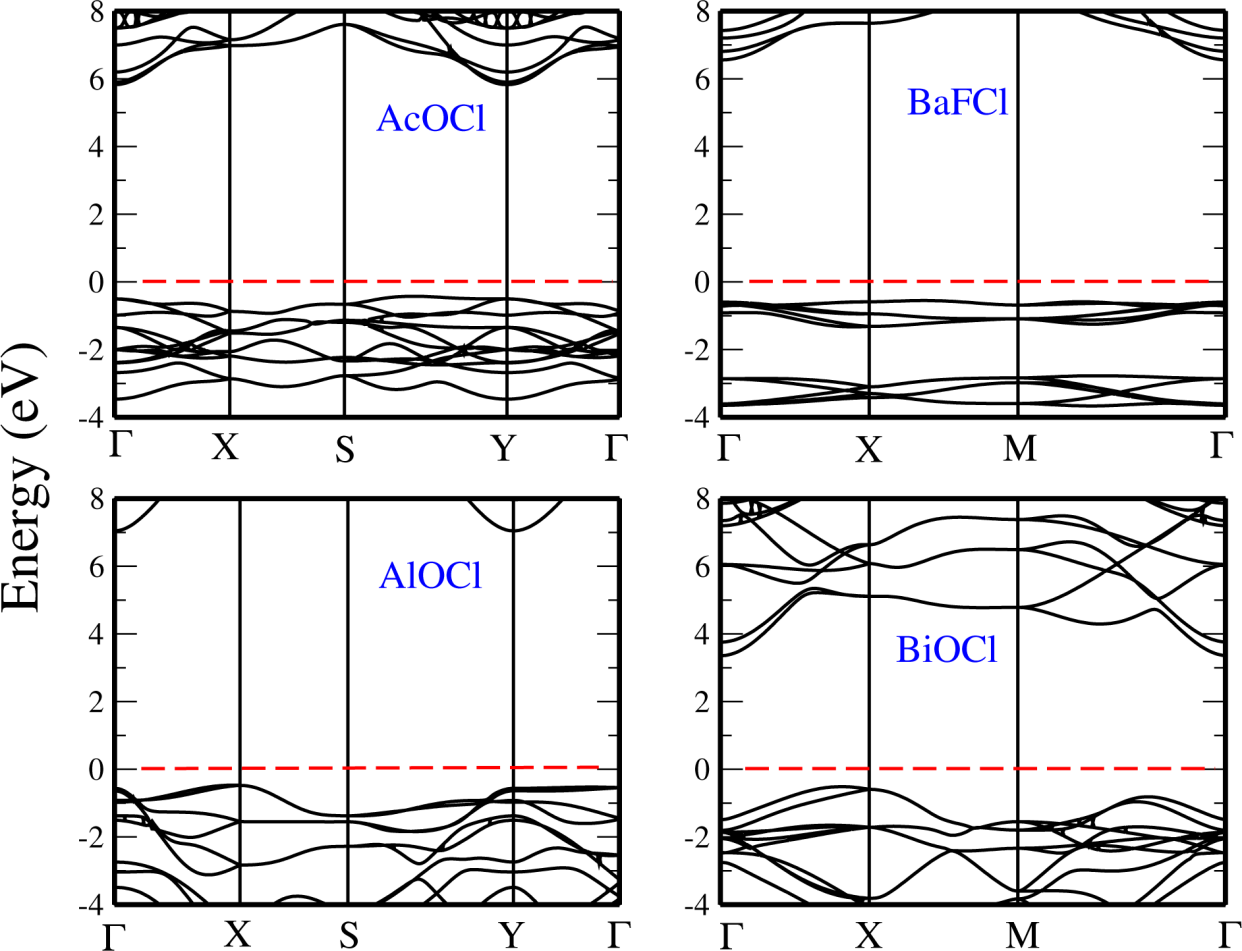
Band structures of the monolayers AcOCl, AlOCl, BaFCl, and BiOCl, using the HSE functional. The Fermi level is set to 0 eV.

We consider now the XOCl and X′FCl (where X = Ac, Al, and X′ = Ba, Bi) monolayers and show their HSE electronic band structures in Figure 9. AcOCl is found to have an indirect bandgap with a value of 6.1 eV with HSE, with the VBM along the S–Y line and the CBM located at the Y point. Next to the band structure of AcOCl, we present the electronic bandstructure of AlOCl. Our results indicate that this monolayer has an indirect bandgap with a value of 7.5 eV (with HSE), with the valence-band maximum and the conduction-band minimum found at the Y and X high-symmetry points, respectively. Similarly to AlOCl, BiOCl has an indirect bandgap with the valence-band maximum located at the X point and the conduction-band minimum at the Γ point. Not surprisingly, the value of the bandgap obtained with the HSE functional is much larger than with the GGA. The values of the bandgap are 2.91 eV with GGA and 4.80 eV with HSE. BaFCl is found to be a direct-gap semiconductor with the valence-band maximum and the conduction-band minimum at the Γ point.

**Figure 10:**
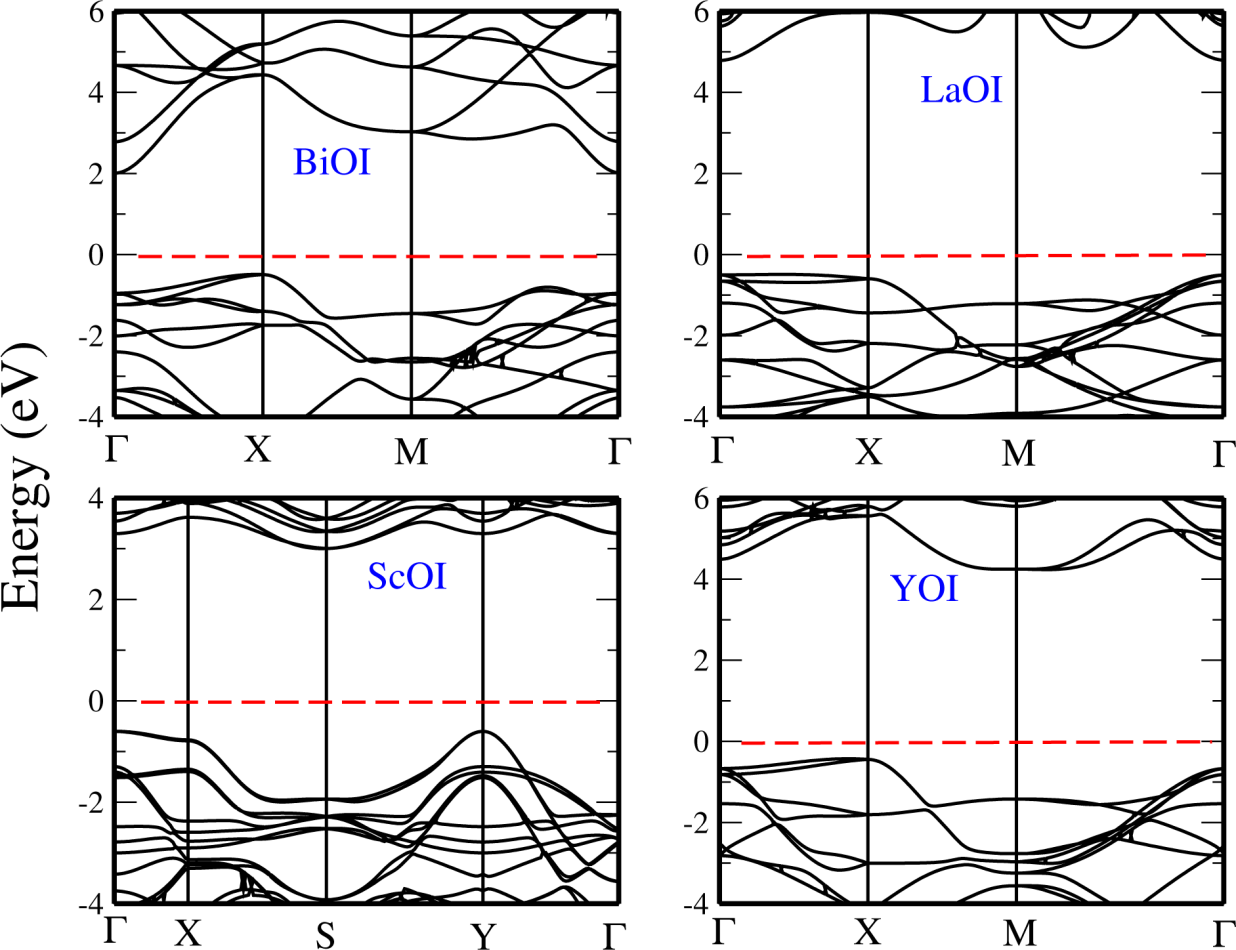
Band structures of the monolayers : BiOI, LaOI, ScOI, and YOI, using HSE functional. The Fermi level is set to 0 eV.

Finally, we have computed the electronic band structures of the XOI monolayers (where X = La, Sc, Y, and Bi), which are presented in Figure 10. We found that the bandgap of BiOI is indirect with the VBM located at the X point, and the CBM at the Γ point, with a value of 1.68 eV with GGA and 3.0 eV with HSE. The monolayers of LaOI and ScOI are found to be direct-bandgap materials at the Γ point, with a HSE bandgap of 5.30 eV and 3.0 eV, respectively. As for the YOI monolayer, we have found it to be an indirect-bandgap compound with the valence-band maximum at the Γ point while the conduction-band minimum is located at the X point, with a HSE value of 5.20 eV for the bandgap.

The values of the bandgaps obtained with the various levels of theory (GGA and HSE) as well as their nature (direct or indirect) are summarized in Table 2. It is seen that the systems studied here offer a wide range of electronic direct or indirect bandgaps, from 3.0 eV for a monolayer of BiOI or of ScOI up to 7.5 eV for a monolayer of AlOCl. Such a bandgap tunability is particularly interesting for the fabrication of flexible and ultrathin optical devices, since it is known from earlier studies [42] that 2D materials can display a much larger sunlight absorption than commonly employed semiconductors. Also, the materials studied here can be employed in heterostructures to complement or replace other large-bandgap 2D materials, such as hexagonal boron nitride, or to dissociate excitons and separate charges if a type-II arrangement [43–44] of the bands can be obtained. Notice that the experimental value of the bandgap for some systems is known [41] in the bulk form but the relatively large difference between our calculated HSE values and the experimental data is linked to the fact that electronic screening is much more efficient in a bulk material, and therefore reduces the value of the bandgap significantly in comparison with the one of the corresponding monolayer.

**Table 2:** Comparison of theoretical bandgap energy *E*_g_ (eV) of different monolayers with experimental values (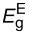) of bulk structure [41]. (D: direct bandgap, I: indirect bandgap).

	bromide monolayers				fluoride monolayers			
	AcOBr	BaFBr	BiOBr	CaFBr	CrOF	GaOF	InOF	LaOF

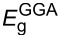	4.57	5.02	2.68	5.17	0.9	3.07	2.13	4.24
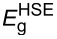	6.1	6.6	4.5	6.9	3.6	5.3	4.0	6.0
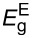 [41]	—	—	2.91	—	—	—	—	—
nature of bandgap	D	D	I	D	—	D	D	I

	chloride monolayers				iodide monolayers			
	AcOCl	AlOCl	BaFCl	BiOCl	BiOI	LaOI	ScOI	YOI

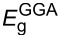	4.60	5.66	5.46	2.91	1.68	3.35	1.67	3.22
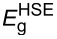	6.1	7.5	7.3	4.80	3.0	5.3	3.0	5.20
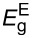 [41]	—	—	—	3.51	1.94	—	—	—
nature of bandgap	I	I	D	I	I	D	D	I

### Effect of an external transverse electric field

Earlier theoretical studies have reported that applying an external electric field to a rippled MoS_2_ monolayer [45] or a MoS_2_ nanoribbon [46–47] causes important changes in the electronic structure and reduces the bandgap. Also, applying an electric field to a 2D material mimics the presence of a gate voltage [48], and understanding the resulting changes in the electronic structure is important for the fabrication of transistors. Therefore, we have used a similar strategy to tune the bandgap of the different monolayers, with electric fields up to 1.0 V/Å applied perpendicularly. A full relaxation of the structure was conducted under the electric field. Notice that the effect of an internal electric field on a slab model of various polar surfaces has been studied in earlier works [49–50].

**Figure 11:**
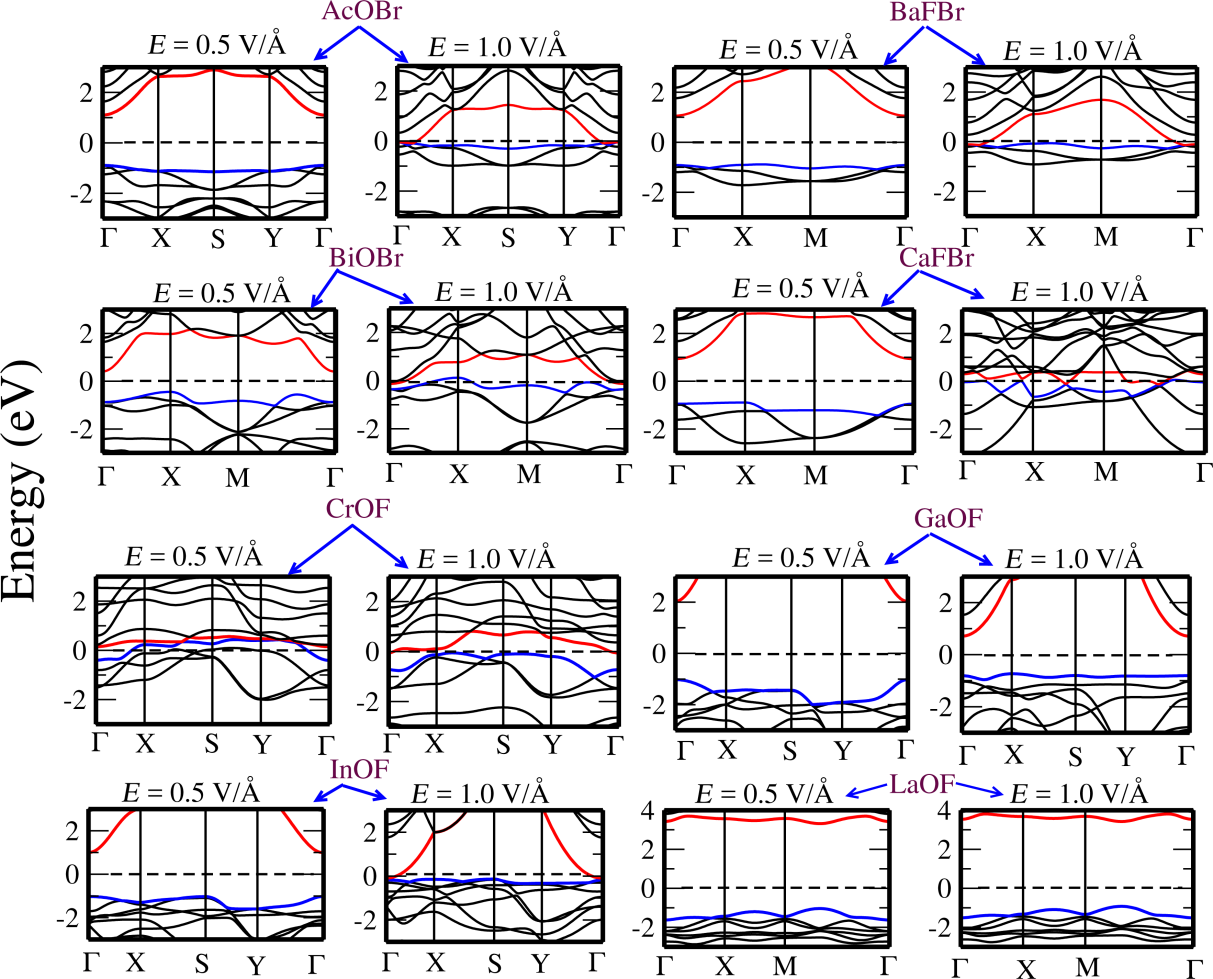
Evolution of the electronic band structure of AcOBr, BaFBr, BiOBr, CaFBr, CrOF, GaOF, InOF, and LaOF single layers as a function of applied electric field. Calculations are performed with PBE. The top of the valence band (red) and bottom of conduction band (blue) are indicated. The Fermi level is set to 0 eV.

**Figure 12:**
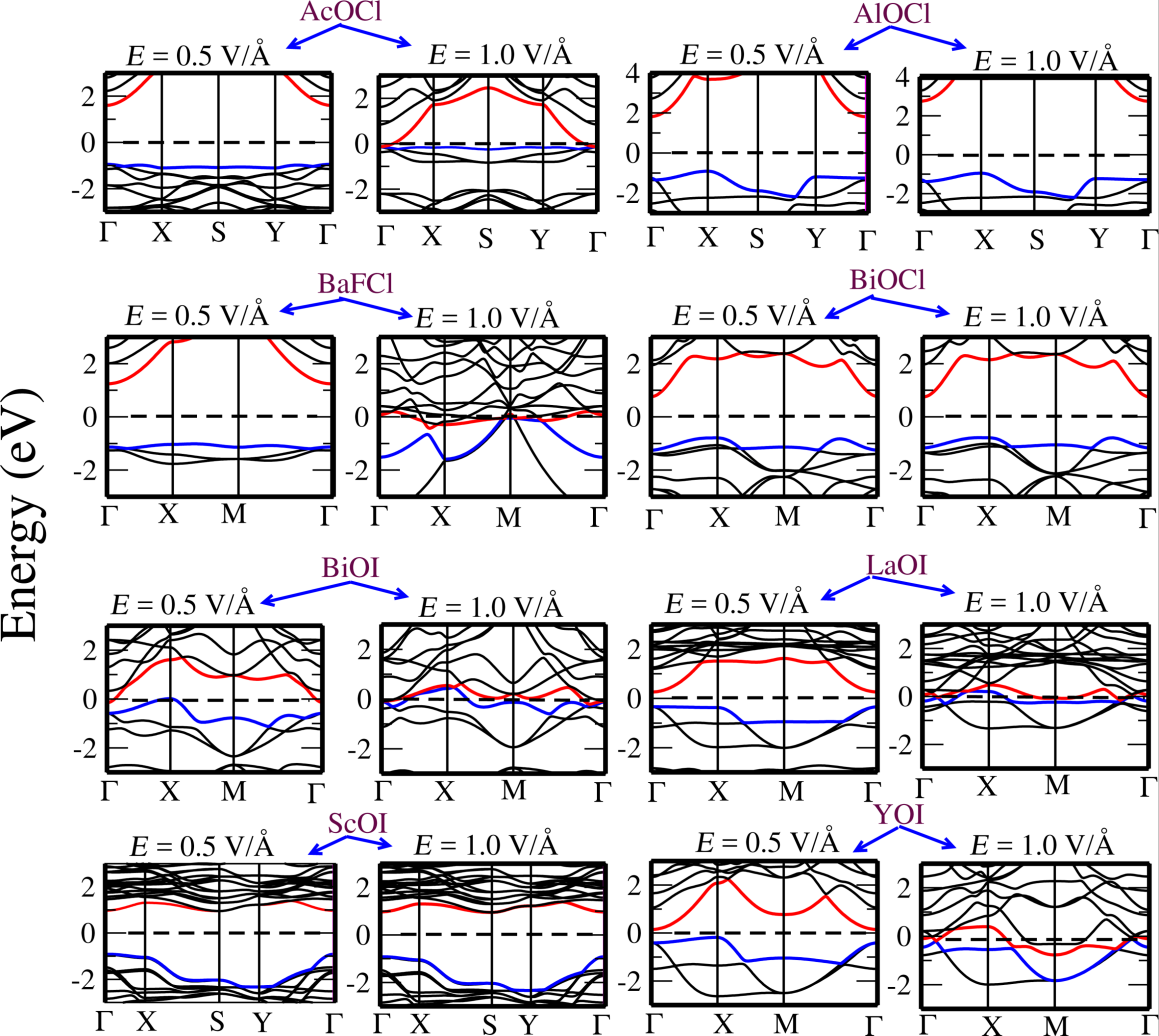
Evolution of the electronic band structure of: AcOCl, AlOCl, BaFCl, BiOCl, BiOI, LaOI, ScOI, and YOI single layer as a function of applied electric field. Calculations are performed with PBE. The top of the valence band (blue) and bottom of conduction band (red) are indicated. The Fermi level is set to 0 eV.

The electronic band structures calculated under an electric field (with values of 0.5 V/Å and 1.0 V/Å) are given in Figure 11 and Figure 12. It can be seen that the effect of the electric field is quite remarkable on some compounds, as for instance a field of 0.5 V/Å can reduce the bandgap of AcOCl from 4.60 eV to 2.08 eV. In a similar way, the bandgap of AcOBr decreases rapidly when increasing the strength of the electric field, resulting in a semiconductor-to-metal transition. A similar phenomenon can be seen for BaFCl, BiOI, and LaOI, where the bandgaps are reduced significantly. However, the bandgap tuning effect is less significant in some other monolayers such as LaOF, BiOCl, and AlOCl, where the application of an external field up to 1.0 V/Å does not modify significantly the value of the bandgap.

Notice that in order to reduce the computational work, our calculations under electric field have been conducted with the GGA functional, which underestimates the bandgap values in comparison with the HSE functional (see Table 2). However, our discussion about the effect of the electric field on the electronic structure still holds, although a larger value of the field would be needed to close the bandgaps obtained with HSE.

## Conclusion

In conclusion, we have calculated the properties of several two dimensional halides compounds using density functional theory. First, we have investigated their electronic structure, and demonstrated their dynamical stability by computing their phonon spectra (see Supporting Information File 1). We have found that their electronic bandgap energies range between 3.0 and 7.5 eV and that CrOF is found to have a spin polarisation. Finally, we have shown that for some of them the value of the bandgap can be decreased by applying an external electric field and even a semiconductor-to-metal transition can be induced. We hope that our work will trigger interest in this family of possible new two-dimensional materials, in particular among experimentalists. Since bulk crystals are known to exist for some of them, mechanical exfoliation can be attempted to isolate one or few layers, and then to explore their electronic and optical properties. If successful, devices such as transistors, based on these monolayers could be fabricated, as it was done with MoS_2_ [51] and phosphorene monolayers [52]. At the same time, the compounds studied could be relevant in optics and optoelectronics, to design new photodetectors, polarizing filters, or modulating devices, as has already been done with other two-dimensional compounds [53–54]. Also, the role of defects and their stability when exposed to air are presently unknown, and can be seen as a direction for future theoretical and experimental studies.

## Supporting Information

File 1 Vibrational properties of the compounds.
