# Supplementary material for: Electronic properties of several two dimensional halides from ab initio calculations
Source: Beilstein J Nanotechnol. 2019 Apr 3;10:823–32. doi: 10.3762/bjnano.10.82 (PMC6466728; doi:10.3762/bjnano.10.82)
Supplement: File 1 — Vibrational properties of the compounds. [file Beilstein_J_Nanotechnol-10-823-s001.pdf]

BEILSTEIN JOURNAL OF NANOTECHNOLOGY

Supporting Information

for

Electronic properties of several two dimensional halides from ab initio calculations

Mohamed Barhoumi, Ali Abboud, Lamjed Debbichi, Moncef Said, Torbjörn Björkman, Dario Rocca and Sébastien Lebègue

Beilstein J. Nanotechnol. **2019,** *10,* 823–832. doi:10.3762/bjnano.10.82

Vibrational properties of the compounds

License and Terms: This is a supporting information file under the terms of the Creative Commons Attribution License (http://creativecommons.org/licenses/by/4.0). Please note that the reuse, redistribution and reproduction in particular requires that the authors and source are credited.
The license is subject to the *Beilstein Journal of Nanotechnology* terms and conditions: (https://www.beilstein-journals.org/bjnano)

We have investigated the vibrational properties of the different monolayers with density functional perturbation theory. The dynamical stability of a monolayer can be demonstrated by calculating its phonon dispersion curve and observing that no imaginary frequency is present. In Figure S1, we present the phonon dispersion curves of the monolayers of AcOBr, BaFBr, BiOBr, and CaFBr. They do not display any imaginary frequency in the spectra, meaning that they are dynamically stable.

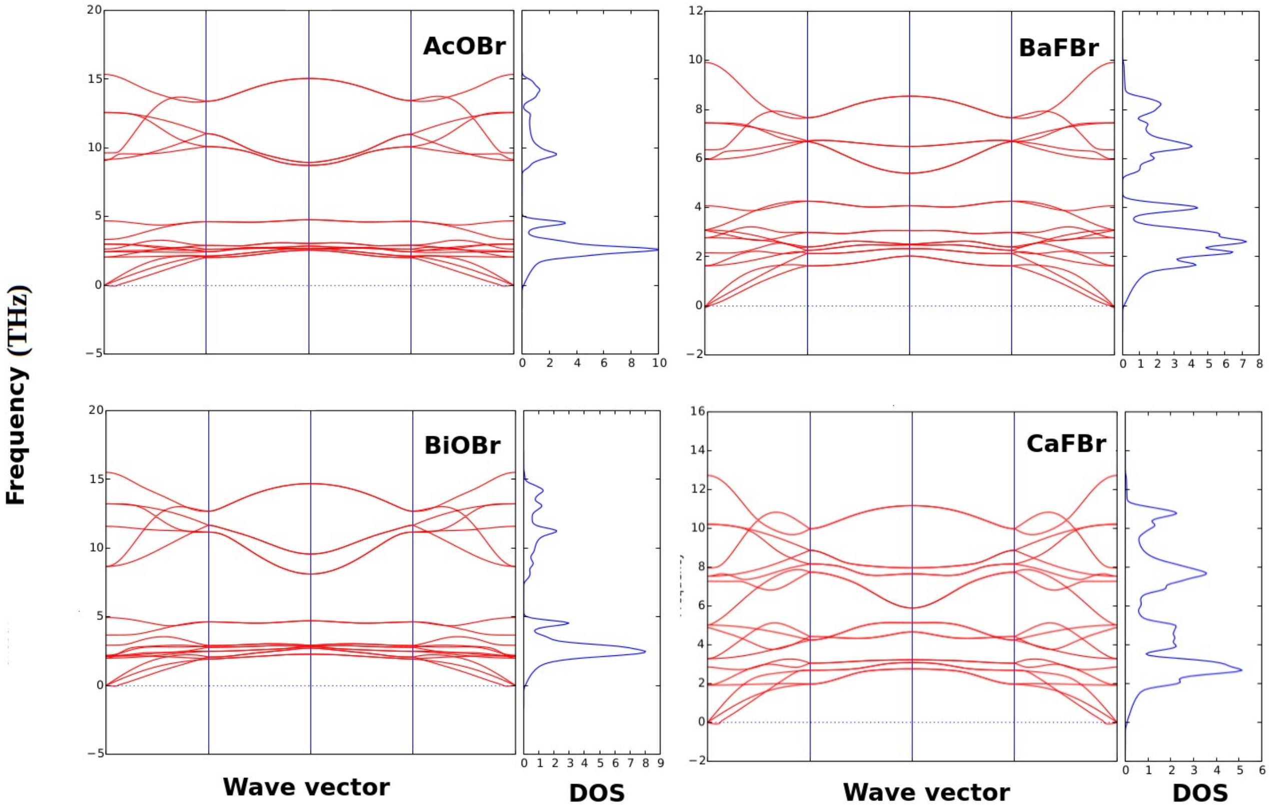

Figure S1: Phonon dispersion curves of the monolayers AcOBr, BaFBr, BiOBr, and CaFBr.

Then, we have computed the phonon dispersion of the fluorides monolayers CrOF, GaOF, InOF, and LaOF (see Figure S2). We have obtained that no imaginary frequency appears in their phonon band spectra.

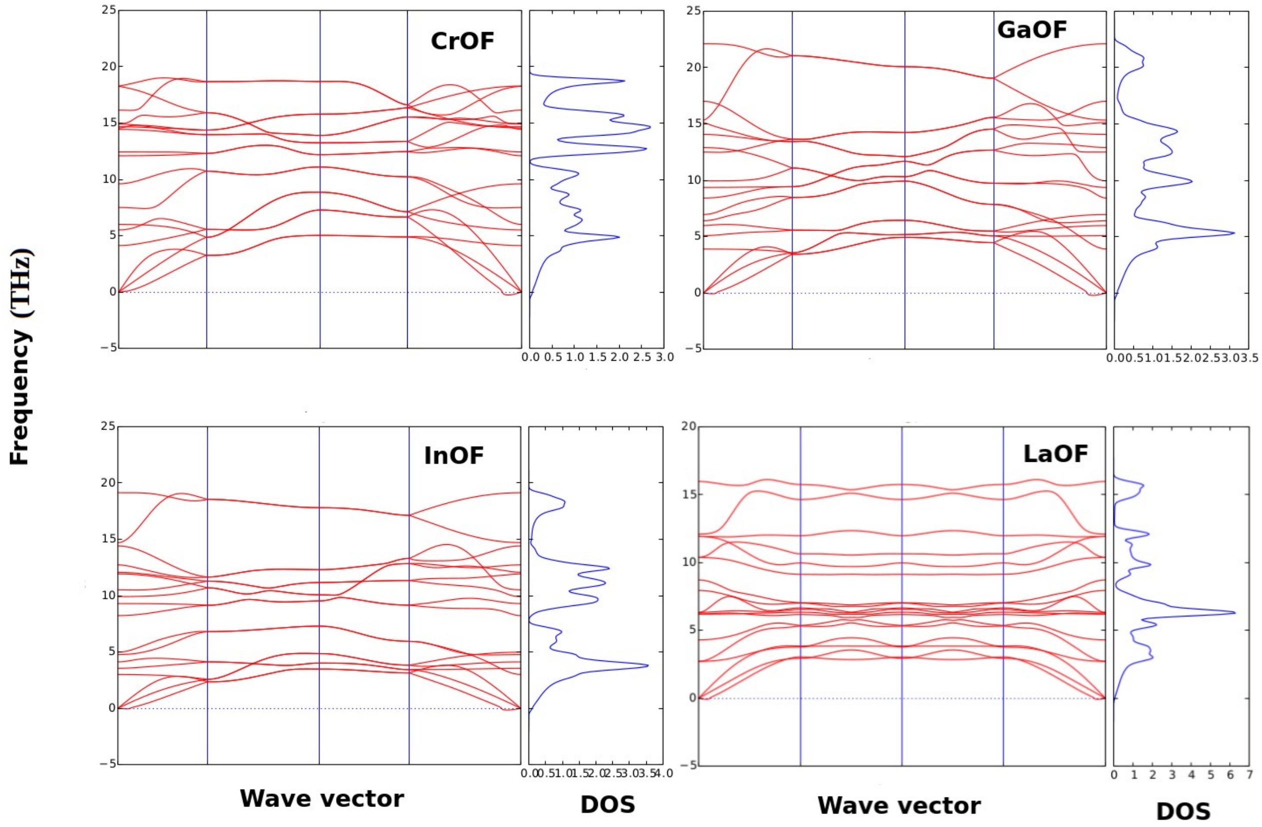

Figure S2: Phonon dispersion curves of the monolayers CrOF, GaOF, InOF, and LaOF.

In the same way, we have studied the dynamical stabilities and the phonon density of states of several chloride monolayers (AcOCl, AlOCl, BaFCl, and BiOCl): Figure S3 illustrates our calculated phonon dispersions for this family of compounds. Here, no imaginary vibration frequency appears in the phonon band spectra for these materials, which indicates that the structures have a vibrational stability.

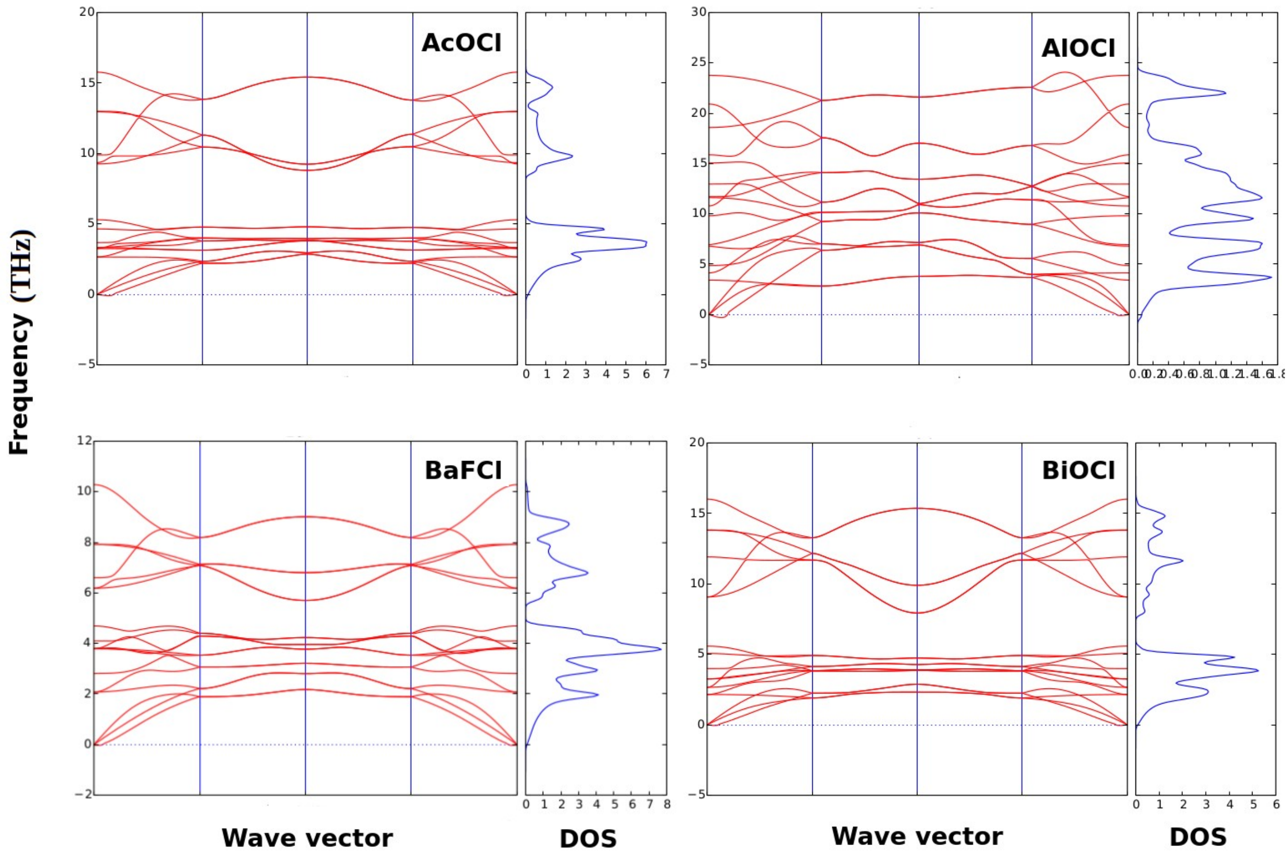

Figure S3: Phonon dispersion curves of the monolayers AcOCl, AlOCl, BaFCl, and BiOCl.

Finally we have turned to the iodides monolayers (BiOI, LaOI, ScOI, and YOI) and present in Figure S4 their phonon dispersion curves. Here also no imaginary frequencies are observed, confirming their dynamical stability when put in a two-dimensional form. Notice that for some cases there is a slightly unstable phonon mode near the Brillouin zone center: this does not correspond to a real instability but is due to the difficulty to achieve a perfect numerical convergence for the phonon spectra 2D materials, which was already commented in previous works of Fal'ko et al [1, 2] on different 2D systems.

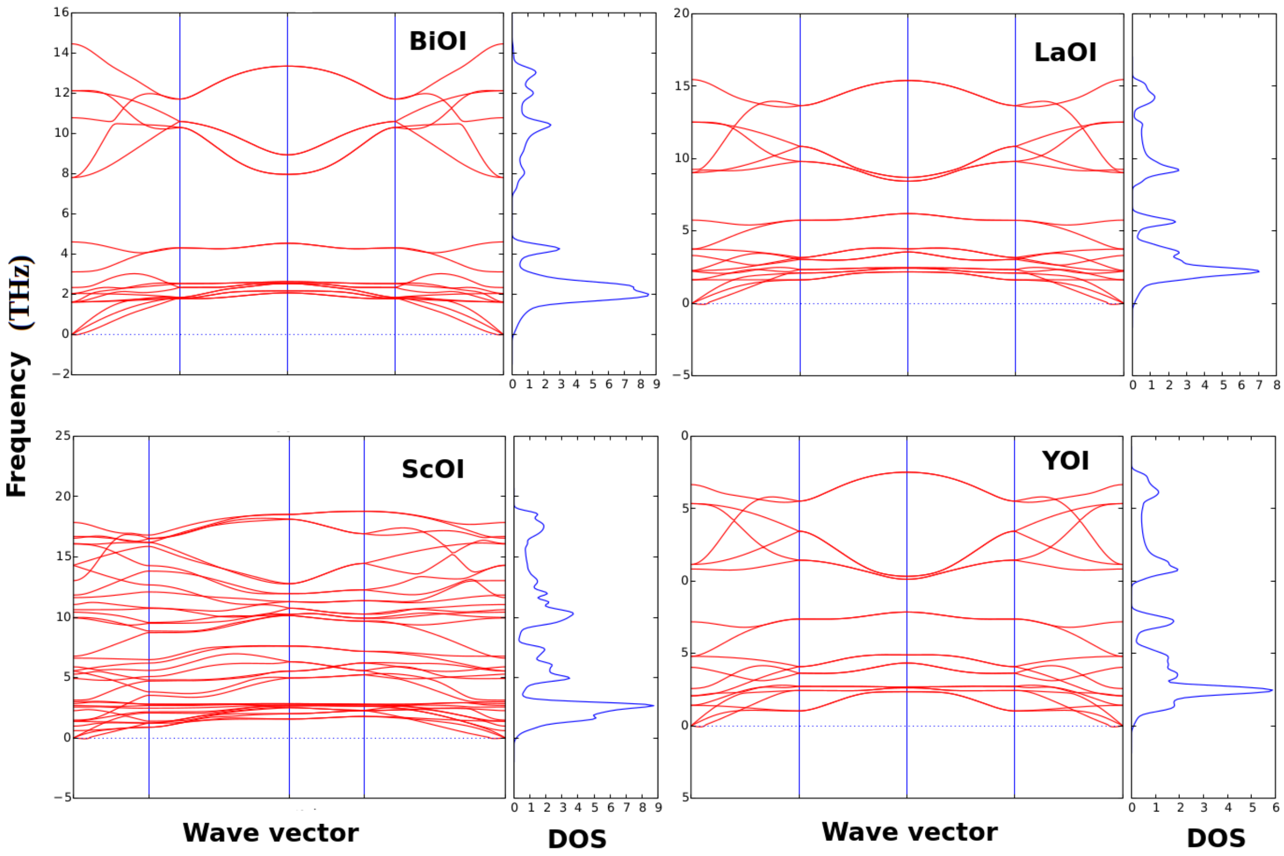

Figure S4: Phonon dispersion curves of the monolayers BiOI, LaOI, ScOI, and YOI.

References

1. Zölyomi, V.; Drummond, N. D.; Fal'ko, V. I. Phys. Rev. B 2014, 89, 205416. doi:10.1103/physrevb.89.205416.

2. Zölyomi, V.; Drummond, N. D.; Fal'ko, V. I. Phys. Rev. B 2013, 87, 195403. doi:10.1103/ physrevb.87.195403.
